# Helping primary care providers recognize and respond to medication non-adherence and drug-drug interactions: A randomized-controlled clinical utility trial in a value-based care setting

**DOI:** 10.1371/journal.pone.0344906

**Published:** 2026-03-16

**Authors:** Trever Burgon, David Paculdo, Joshua Schrecker, Kelsy Gibson Ferrara, Randy E. David, Steven Johnson, Czarlota Valdenor, John W. Peabody

**Affiliations:** 1 QURE Healthcare, Saint Louis, Missouri, United States of America; 2 Aegis Sciences Corporation, Nashville, Tennessee, United States of America; 3 Wayne State School of Medicine, Detroit, Michigan, United States of America; 4 University of California, San Francisco, California, United States of America; 5 University of California, Los Angeles, California, United States of America; Ehime University Graduate School of Medicine, JAPAN

## Abstract

**Objective:**

To determine the clinical utility of a novel chronic disease management test among primary care providers practicing in a value-based care setting. The study assessed the ability of primary care providers to recognize and respond to medication nonadherence (MNA), drug-drug interactions (DDIs), and disease progression.

**Design:**

A pre-post experimental randomized controlled trial of primary care providers in the same practice setting caring for simulated patients with chronic disease.

**Setting and participants:**

139 licensed primary care providers (MD, DO, NP, or PA) at a large, multi-state primary care practice in the United States.

**Intervention:**

Both intervention and control group participants cared for three simulated chronic disease patients at baseline and again after the intervention. After baseline, intervention group participants reviewed educational materials about a new saliva-based chronic disease management test that detects MNA and DDI and were then provided with the test’s results in simulation; the control group did not have access to the test results.

**Primary outcome measures:**

Changes in care decisions between the two groups were analyzed using a difference-in-differences approach to determine whether routine testing in the outpatient setting changed correct identification and management of medication nonadherence (MNA), drug-drug interactions (DDI), and disease progression.

**Results:**

The intervention group participants were significantly more likely to make the correct diagnosis of MNA (control: 10.0% vs intervention: 80.0%, p < 0.001) and DDI (control: 18.6% vs intervention: 52.9%, p < 0.001) and to recommend an appropriate treatment for MNA (control: 25.7% vs intervention: 54.3%, p = 0.001) and DDI patients (control: 27.1% vs intervention: 58.8%, p < 0.001).

**Conclusion:**

Primary care providers with results from a definitive saliva-based test for MNA and DDI were significantly more likely to identify those common challenges and make appropriate updates to the patient’s treatment plan. This has important implications for improving quality and value, especially for the millions of patients with chronic conditions on multiple medications.

**Trial registration:**

ClinicalTrials.gov, NCT05658653, Registered 2-Dec-2022.

## Introduction

Patients with chronic diseases and worsening symptoms are challenging to manage in primary care [1]. A typical management paradigm is to assess the patient’s symptoms and adjust or change medications as necessary. This may be combined with additional laboratory tests, imaging, or referral to a specialist. If these steps are taken without carefully considering medication nonadherence (MNA) or drug-drug interactions (DDIs), physicians and other primary care providers (PCPs) may erroneously attribute worsening symptoms to disease progression [2]. These challenges are especially relevant for organizations in value-based care payment models, where efficient work-up (e.g., avoid unneeded testing) and effective management that reduces the risk of adverse events and hospitalizations are key to success [3].

The clinical impacts of polypharmacy, MNA, and DDIs have been well documented and include reductions in health-related quality of life [4], increases in adverse drug events [5], hospitalizations [6,7], and billions of dollars in healthcare expenditures [8]. A study of four adult primary care practices in the US found that 63% of ameliorable adverse drug events were attributable to the provider’s failure to respond to medication-related symptoms [9]. The lack of simple, objective testing options to detect MNA and DDIs makes it difficult to consistently identify and address these issues, as evidenced by a practice assessment that found that while 99% of practitioners claim that they perform effective medication reconciliation to identify MNA, only 3.3% do [2]. PCP assessments of adherence only aligned with objective pharmacy refill assessments approximately 50% of the time [5]. Furthermore, we found that only 8.2% of practitioners checked for DDIs [2], a potentially severe issue underscored by the ubiquity of polypharmacy in the outpatient setting. Using a common definition of polypharmacy as the concomitant use of five or more medications [10], the prevalence of polypharmacy has been continually increasing among older adults in the United States, from 24% between 1999–2000 to 44% between 2017–2018 [11]. An objective test to identify potential MNA and DDIs in patients diagnosed with common chronic diseases would help providers formulate appropriate treatment plans and improve health outcomes. Once MNA and DDI are excluded as causes for worsening symptoms, disease progression can be assessed and managed appropriately.

The study team partnered with a large multi-state primary care practice that identified chronic disease management as a priority for quality improvement and success in value-based care, recognizing the challenges that MNA and DDIs pose among their patient population. The practice was particularly focused on improving heart failure (HF) and chronic obstructive pulmonary disease (COPD) care, two of the most common and costly chronic outpatient conditions in the US [12–15]. A systematic review of 22 studies of HF patients showed that rates of polypharmacy ranged from 17.2% to 99% [16], and a separate study showed that rates ranged from 22% to 30% among patients with COPD [17]. Providing the right medication at an appropriate dose for patients with these conditions can make a sizable impact on outcomes; evidence-based drug treatment has been shown to lead to a 68% reduction in heart failure with reduced ejection fraction (HFrEF) hospitalizations and a 22% reduced risk of COPD exacerbations [18–21].

As a follow-up to our national study of the clinical utility of this novel, saliva-based chronic disease management test among primary care physicians [22], we conducted this randomized controlled trial within a single practice committed to value-based care among both primary care physicians and advanced-practice providers (APPs). We asked primary care providers to care for virtual patient simulations with HF and COPD in a pre-post experimental design, where the intervention group was provided with results from the saliva-based test in the virtual cases. The control group did not have access to the test results. We compared the change in practice between the two groups using a difference-in-differences calculation to determine if routine testing in the outpatient setting changed the way patient follow-up visits were managed.

This randomized controlled trial provides insight into the clinical value and utility of a novel chronic disease management test within a large, value-based-care-focused multistate primary care practice in the United States. The use of scientifically validated virtual patient cases allowed for standardized assessment of clinical decisions across the test’s various clinical indications, and the inclusion of advanced practice providers (NPs and PAs) in the study sample allowed for the comparison of chronic disease management decisions between provider types.

## Methods

### Overview

Beginning August 17, 2022, and running between August 2022 and December 2022, 150 primary care providers within a large, multi-state practice were recruited to voluntarily participate in a randomized controlled trial (RCT) by caring for online, virtual patients. The study assessed the clinical utility of the novel chronic disease management test by measuring the ability of providers to recognize and respond to disease progression, MNA, and DDIs with and without the novel test results.

### Ethics

This study was conducted in accordance with ethical standards and approved by the Advarra Institutional Review Board, Columbia, MD, USA, in June 2022 prior to the start of the study, and subsequently listed on ClinicalTrials.gov (NCT05658653) in December 2022 after study commencement due to organizational oversight. Voluntary informed consent was obtained from all participants before the study commenced.

### Chronic disease management test

Aegis Sciences Corporation [23] recently developed a novel, commercially available, chronic disease management test (MedProtect CDM^®^) that detects MNA and DDI. Testing is performed on saliva specimens that can be non-invasively collected in an outpatient office setting. The chronic disease management test (CDMT) is capable of detecting >150 drugs and metabolites including the most common medications for managing cardiometabolic disease, including angiotensin-converting enzyme inhibitors, angiotensin receptor blockers, beta-blockers, diuretics, statins, sulfonylureas, antithrombotic agents, inhaled medications (such as beta-agonists, anticholinergics, and corticosteroids) as well as non-prescribed drugs (including over the counter medications and supplements) [24].

### Participant sample

Potential primary care provider participants within a single practice committed to value-based care were identified via a roster provided by practice leadership. Potential participants were contacted by email using a recruitment letter.

We serially enrolled the first 150 practicing primary care providers (physicians and advanced practice providers [nurse practitioners and physician assistants]) who met eligibility criteria, including:

- licensed primary care provider (MD [Doctor of Medicine], DO [Doctor of Osteopathic Medicine], NP [Nurse Practitioner], or PA [Physician Assistant]) currently practicing in General Internal Medicine or Family Medicine
- in practice as a licensed primary care provider for greater than two but less than 30 years
- currently caring for 40 or more patients weekly
- commonly treating patients with primary diagnoses of congestive heart failure and/or COPD and common comorbidities, including diabetes, hypertension, and atrial fibrillation
- practicing in the US
- English-speaking
- have access to broadband internet
- voluntarily consented to be a study participant.

These 150 primary care providers were randomized to about 75 participants per study arm. These numbers were chosen to have sufficient power to detect an effect size of 6% on the overall score between the two study arms.

### Data sources

This study included two primary sources of data: a participant questionnaire and the providers’ decisions as they cared for the virtual patient cases. The questionnaire probed demographics, details of medical practice, location of practice, practice type, and patient make-up, among other items. The virtual patient cases evaluated clinical practice decisions.

### Virtual patient cases

QURE’s virtual patient cases are a validated methodology, widely used to rapidly measure physician care decisions [25–29]. Virtual patients use open-ended questions simulating typical patient encounters, with questions divided into five domains of care: (1) medical history taking, (2) physical examination, (3) diagnostic work-up, (4) making a diagnosis, and (5) management plan and patient monitoring.

We created nine virtual patient cases in a matrix with 3 case types: (1) heart failure with reduced ejection fraction (HFrEF), (2) heart failure with preserved ejection fraction (HFpEF), and (3) COPD. See ‘S1 Appendix’ Word document for case matrix and patient details. All patients had comorbid conditions such as diabetes mellitus, chronic kidney disease, depression, etc., and were symptomatic at their outpatient visits. Each case type had three variants (A) MNA no DDI; (B) DDI no MNA; (C) No MNA nor DDI; disease progression likely (Appendix). In both rounds of the study, participants completed three virtual patient cases, one from each case type and case variant, in random order. To assess their baseline practice patterns in the first round of cases, both control and intervention participants completed their cases without access to the CDMT test results measuring MNA or DDI. In the second round of cases, intervention participants now had CDMT results unlocked as they cared for their next three cases, while control participants only had access to standard-of-care tools and were not offered CDMT test results.

Responses of each participant were independently scored by two trained physician experts using predetermined criteria based on current standards of care (Cohen’s kappa, 0.77 to 0.81). In the case of disagreement in physician scoring, a third physician served as an adjudicator.

### Intervention

Providers were block-randomized (2–6 participants per block) using a blind coin flip methodology to either the control or intervention arm of the study. After completion of the first (baseline) round of virtual patients where no participant had access to the test, intervention group providers were provided educational materials on the CDMT. Education materials included a slide deck, fact sheet, and case studies and were designed to orient the participant to test specifications and interpretation of test results. After viewing educational materials, both intervention and control group participants completed their second round of virtual patients.

### Outcome measures

The primary outcome measure was the recognition of MNA, DDI, or disease progression as the underlying cause of symptoms in the respective cases. We contrasted differences in MNA, DDI, and disease progression identification made by intervention versus control providers. More specifically, we evaluated whether the CDMT results improved the identification of MNA/DDI and subsequent provision of an evidence-based primary care management plan for each case variant. Correct management was defined as: continuing medications or providing medication counseling in the case of MNA, stopping at least one of the interacting medications in the case of DDI, or increasing medications, performing further workup, or initiating other interventional procedures for disease progression. Our secondary outcome was to determine whether there was a difference in improvements between physicians and APPs when given the results of the CDMT.

### Statistical analysis

Summary statistics were calculated for all variables. All primary and secondary endpoints were binary (correct vs incorrect), and percentages presented in tables and figures are descriptive sample proportions.

We evaluated intervention effects using a difference-in-differences approach, defined as the change from baseline to follow-up in the intervention arm minus the change from baseline to follow-up in the control arm. For binary endpoints, we estimated unadjusted logistic regression models with indicators for study arm (intervention vs control), time (Round 2 vs Round 1), and an arm-by-time interaction term. The arm-by-time interaction term was used to test the difference-in-differences effect. Because each participant contributed multiple case observations, standard errors were clustered at the participant level [30–32].

Where shown, p-values for between-arm comparisons within a given round were obtained using chi-square tests on the underlying binary outcomes. Variant-level and other secondary analyses are exploratory; p-values are unadjusted and interpreted cautiously. All analyses were conducted using Stata 18.0 software.

## Results

### Participant characteristics

**Fig 1:**
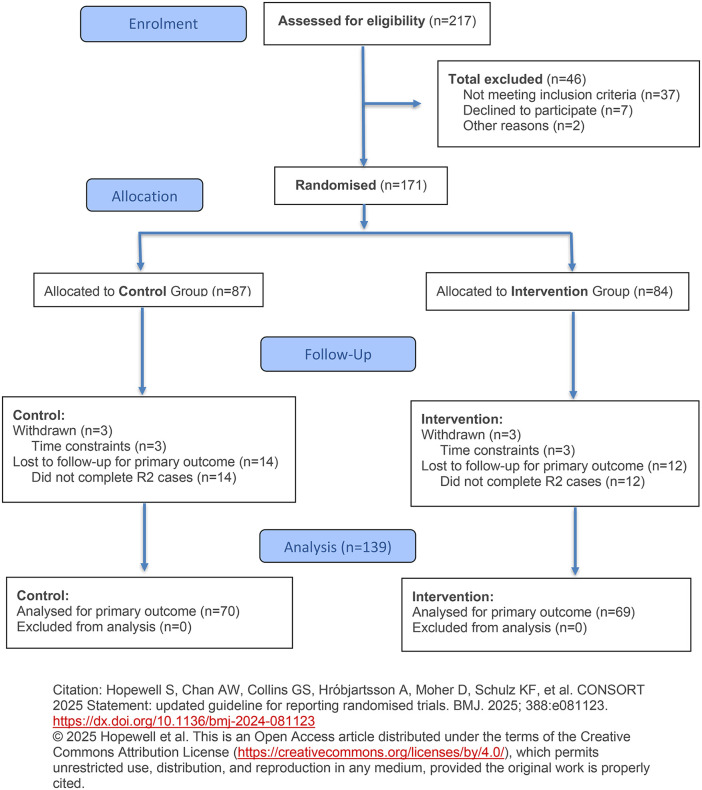
Flowchart of potential participants through the randomized trial.

One hundred and fifty individuals completed the first round of data collection and the provider survey (Fig 1). Of these, 11 reported that they could not complete the second round of data collection due to time constraints. In all, 139 participants completed all six virtual patient cases across the two rounds (Table 1). There were no significant differences in participant characteristics between the intervention and control arms of the study. Approximately 30% of participants identified as male and 70% as female in both groups (p = 0.608), and approximately 70% were advanced practice providers (APPs) (p = 0.892), with the remainder being physicians. We found no statistically significant differences between control and intervention arms based on any tracked variable (p > 0.05 for all).

**Table 1:** Baseline participant characteristics.

	Control	Intervention	P value
N	70	69	--
Gender			
Male	30.0%	26.1%	0.608
Female	70.0%	73.9%	
**APP**	71.4%	72.5%	0.892
Age Group			
<40	30.0%	33.3%	0.293
40-55	48.6%	36.2%	
>55	21.4%	30.4%	
Practice Setting			
Urban	33.8%	27.9%	0.683
Suburban	58.8%	61.8%	
Rural	7.4%	10.3%	
Region			
Northeast	11.8%	10.3%	0.774
South	58.8%	54.4%	
Midwest	11.8%	10.3%	
West	17.7%	25.0%	
Payer			
Medicare	39.5%	39.3%	0.959
Medicaid	9.8%	8.2%	0.480
Commercial	42.4%	46.0%	0.248
Self	6.9%	6.1%	0.444
Other	1.3%	0.3%	0.077
**Employed by Practice**	98.5%	95.6%	0.310
**Participant in** **CMS** **Quality Program**			
Yes	58.8%	50.0%	0.506
No	7.4%	11.8%	
Don’t know	33.8%	38.2%	

### Baseline identification of the principal cause of presenting symptoms

At baseline, participants correctly identified the cause(s) of their patients’ symptoms in 35.3% of cases. In round one, there was no statistically significant difference between study arms (control: 35.7% vs intervention: 34.8%, p = 0.842) (Table 2), regardless of the primary cause of symptoms (MNA, DDI, or disease progression). Notably, less than 25% of participants correctly identified MNA in the MNA-caused cases (Variant A: control: 18.6% vs intervention: 16.4%, p = 0.740) or DDI in the DDI-caused cases (Variant B: control: 21.1% vs intervention 17.4%, p = 0.575). However, more than two-thirds identified disease progression in the disease progression-caused cases (Variant C: control: 68.1% vs intervention 69.0%, p = 0.909).

**Table 2:** Correct identification of reason for presenting symptoms, overall and by case variant. Variant-level analyses are exploratory and p-values are unadjusted. Round-specific p-values compare study arms within a round using chi-square tests. Difference-in-differences p-values test the arm-by-time interaction from the regression model.

All Cases	Round 1	Round 2	P value	Difference-in-Differences	Difference-in-Differences, P value
Control	35.7%	32.9%	0.537		
Intervention	34.8%	69.6%	<0.001	37.6%	<0.001
P value	0.842	<0.001			
**Variant A – Medication Non-Adherence**	Round 1	Round 2	P value	Difference-in-Differences	Difference-in-Differences, P value
Control	18.6%	10.0%	0.147		
Intervention	16.4%	80.0%	<0.001	72.2%	<0.001
P value	0.740	<0.001			
**Variant B – Drug-Drug Interaction**	Round 1	Round 2	P value	Difference-in-Differences	Difference-in-Differences, P value
Control	21.1%	18.6%	0.704		
Intervention	17.4%	52.9%	<0.001	38.1%	0.002
P value	0.575	<0.001			
**Variant C – Disease Progression**	Round 1	Round 2	P value	Difference-in-Differences	Difference-in-Differences, P value
Control	68.1%	70.0%	0.810		
Intervention	69.0%	75.4%	0.402	4.5%	0.665
P value	0.909	0.478			

### Impact of CDMT on diagnosis of MNA, DDI, and disease progression

**Fig 2:**
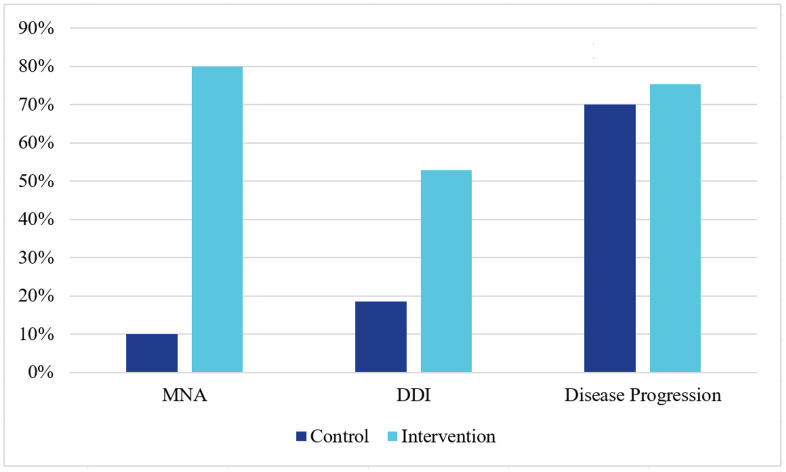
Percent of participants, by study arm, that correctly identified medication nonadherence (MNA), drug-drug interactions (DDI), and disease progression in the second round. Percentages are descriptive proportions of underlying binary outcomes. Inferential results (including difference-in-differences tests) are reported in Table 2. Variant-level analyses are exploratory and p-values are unadjusted.

After introducing the CDMT to the intervention group, we compared clinical practice in the second round of data collection (Table 2 and Fig 2). The intervention group was significantly more likely to appropriately diagnose MNA in the variant A cases in round 2 (control: 10.0% vs intervention: 80.0%, p < 0.001), demonstrating a difference-in-differences improvement of 72.2% (p < 0.001). They were also significantly more likely to make the diagnosis of DDI in the variant B cases in the second round (control: 18.6% vs intervention: 52.9%, p < 0.001), again showing a significant difference-in-differences improvement of 38.1% (p = 0.002). There was, however, no significant difference between the intervention and control groups in making the diagnosis of disease progression, either in round 2 or across both rounds (difference-in-differences: + 4.5%, p = 0.665).

### Improved treatment plans with correct diagnosis

Providing CDMT results significantly increased providers’ rates of appropriately continuing medications or providing medication counseling in the MNA cases (difference-in-differences = +25.8%, p = 0.036) and discontinuing interacting medication(s) in the DDI cases (difference-in-differences = +28.3%, p = 0.039) but, surprisingly, did not affect the primary endpoints of deciding to increase medication dose/pursue further intervention in the disease progression cases (difference-in-differences = −20.8%, p = 0.082) (Table 3). When we examined counseling patients on the underlying reason for their symptoms in the DDI and disease progression cases, we saw no significant difference (DDI difference-in-differences = +1.5%, p = 0.711; disease progression difference-in-differences = −7.3%, p = 0.535).

**Table 3:** Correct treatment of and advising on primary cause of symptoms, overall and by case variant. Variant-level analyses are exploratory and p-values are unadjusted. Round-specific p-values compare study arms within a round using chi-square tests. Difference-in-differences p-values test the arm-by-time interaction from the regression model.

All Cases	Round 1	Round 2	P value	Difference-in-Differences	Difference-in-Differences, P value
Control	30.0%	35.7%	0.213		
Intervention	36.7%	53.1%	0.001	10.7%	0.156
P value	0.152	0.001			
**Variant A – MNA (Continue Medications or Provide Medication Counseling)**	Round 1	Round 2	P value	Difference-in-Differences	Difference-in-Differences, P value
Control	27.1%	25.7%	0.846		
Intervention	29.9%	54.3%	0.004	25.8%	0.036
P value	0.714	0.001			
**Variant B – DDI (Stop Interacting Medications)**	Round 1	Round 2	P value	Difference-in-Differences	Difference-in-Differences, P value
Control	21.2%	27.1%	0.402		
Intervention	24.6%	58.8%	0.002	28.3%	0.039
P value	0.631	<0.001			
**Variant C – Disease Progression (Revascularization, HTN Workup; Increase Medications)**	Round 1	Round 2	P value	Difference-in-Differences	Difference-in-Differences, P value
Control	42.0%	54.3%	0.150		
Intervention	54.9%	46.4%	0.314	−20.8%	0.082
P value	0.128	0.349			

Next, we looked at providers in both arms who originally listed an incorrect diagnosis in the baseline round for each case variant (MNA, DDI, or disease progression) but subsequently identified the correct diagnosis in the second-round cases. Perhaps unsurprisingly, these participants only indicated the correct primary treatment 29.6% of the time in the baseline round (when they provided an incorrect diagnosis), compared to 67.2% of the time in the second round (when they provided the correct diagnosis) (p < 0.001). This remained significant across all case variants (MNA baseline: 32.7% vs. second round: 51.9%, p = 0.047; DDI baseline: 19.5% vs. second round: 80%, p < 0.001; disease progression: baseline: 37.5% vs. second round: 75.8%, p = 0.002).

### Comparing physician and APP performance in the intervention arm

When we investigated the performance of advanced practice providers (APPs) vs physicians, at baseline, physicians were about as likely as APPs to correctly identify the cause of their patients’ symptoms (O.R. 1.3, 95% C.I. 0.7–2.4) and provide the correct primary treatment (O.R. 0.7, 95% C.I. 0.4–1.2). This trend was consistent across all three case variants. After the introduction of the CDMT results, intervention-arm physicians showed somewhat greater improvement than intervention-arm APPs in identifying the underlying causes of symptoms (O.R. 1.7), although this was nonsignificant (95% CI 0.7–4.0). Intervention-arm physicians also had higher rates of ordering the appropriate primary treatment (O.R. 1.9, 95% C.I. 1.0–3.7) across all case variants after the introduction of the CDMT. Physicians performed slightly worse across all cases at counseling patients on the cause of their symptoms (O.R. 0.6), although this also was nonsignificant (95% C.I. 0.3–1.6).

## Discussion

Chronic disease management is a consequential challenge facing primary care providers, especially in value-based care arrangements where clinical and financial success is tied to keeping patients healthy and out of the hospital. When patients present with worsening symptoms, it is incumbent on providers to investigate common causes of their symptoms, including medication nonadherence and drug-drug interactions. In this randomized controlled trial, we found that PCPs relying on standard-of-care approaches struggle to identify MNA and DDI challenges among HF and COPD patients, consistent with previous studies [2,22,33]. Our baseline data demonstrated that providers correctly identified MNA, DDI, or disease progression in only one-third of all patient cases, regardless of provider type (physician or APP). However, access to the results from a simple saliva-based test that detects MNA and DDIs markedly increased the rate of correctly identifying MNA and DDI. Disease progression was diagnosed at a similarly high rate by both groups at baseline and post-intervention, strongly suggesting that the novel test results were specifically relevant for MNA and DDI cases rather than general improvements in quality.

Published estimates of drug-drug interaction (DDI) prevalence and medication nonadherence (MNA) in chronic disease populations provide an important epidemiologic context, but clinicians’ perceived likelihood of DDIs and MNA during routine follow-up visits may differ from these published base rates. In busy primary care settings, providers may underweight the probability that new or worsening symptoms are driven by DDIs or nonadherence and instead attribute symptoms to disease progression or other causes. Objective testing may therefore improve care in part by shifting clinicians’ perceived probability of DDIs and MNA in the presenting patient and prompting reconsideration of the differential diagnosis and treatment plan.

Critically, the improvements in the diagnosis of MNA and DDI lead to specific actions to improve patient care. Participants who correctly identified the primary cause of symptoms were significantly more likely to recommend a relevant treatment plan, notably, stopping medications with clinically significant DDIs or continuing medications/counseling patients about MNA. These targeted interventions, when applied regularly in practice, have the potential to deliver significant clinical utility for patients experiencing MNA and DDI and improve quality and efficiency in a value-based care environment. One surprise was the lack of improvement in the intervention group in providing the correct treatment for the disease progression cases. Having been assured through the CDMT results that the cases were not MNA- or DDI-related, we would have assumed that providers would have been more confident recommending actions to address their worsening disease status. That only about half of providers selected an evidence-based treatment plan for the disease progression cases, suggests an opportunity for additional training on care recommendations for heart failure and COPD patients whose disease is progressing. However, some of the change seen in these cases was marked by an unusually high difference between the two groups at baseline which may have been driven by some combination of underperformance in control and overperformance in intervention.

APPs and physicians performed similarly at baseline and after the intervention in identifying MNA and DDI in symptomatic patients. APPs were more likely to identify patient counseling and education as relevant steps to address patient needs, while physicians were more likely to recommend the primary treatment. While further research needs to be done to determine the reasons behind these differences, these results suggest that MNA and DDI testing helps both physicians and APPs make better diagnostic and treatment decisions for heart failure and COPD patients experiencing these challenges.

While the study offers unique insights into the challenges physicians and APPs in value-based care have in diagnosing and responding to MNA and DDIs, it has several limitations and highlights key questions for additional investigation. While the presented cases of chronic cardiometabolic conditions were of particular interest to the participating primary care system, the nine cases used in this study are not a comprehensive presentation of medication adherence and DDI challenges for patients with chronic diseases. Thus, the generalizability of this test to other conditions, such as hypertension and diabetes, is not within the scope of this study’s findings, although this has been shown in other research [34]. Additionally, this study focused on PCP decision-making and did not collect patient-level data. Although this patient simulation-based approach has been validated against actual practice [25–29], additional research was conducted to address this limitation by collecting real-world clinical decision-making data among providers using the CDMT on patients in their practice [34]. The findings of the patient-level study, which demonstrated improvements in relevant clinical actions secondary to utilization of the CDMT, are in alignment with the impact seen in the patient simulation-based approach [31]. Finally, this study sought to address whether testing patients for MNA and DDI provided utility to primary care providers; questions about particular limitations of the test (e.g., pharmaceuticals not included in the test library, lack of personalized genetic information), underlying reasons why patients would become nonadherent to their medication regimen, or why providers might not recognize the signs and symptoms of DDI were outside the scope of the study.

These findings likely have broad clinical implications for chronic disease management in the primary care setting, where providers have many competing priorities and finite time with each patient [35]. A thorough review of a patient’s medication regimen to explore MNA and DDI challenges should be an essential part of a medical evaluation, but solutions are limited, not standardized, and can be time-consuming and/or unreliable [22,36], especially for patients with multiple conditions and polypharmacy. There is a conspicuous need for a definitive tool to help providers identify MNA and DDI challenges facing patients so they can respond with appropriate changes to the management plan. An easily administered, office-based test to definitively identify MNA and DDI among patients with chronic diseases can deliver clinical utility by helping providers recognize and respond to challenges that too frequently go unnoticed and unaddressed today.

## Conclusion

Identification of medication nonadherence and drug-drug interactions is one of many clinical challenges that providers face when treating chronic disease patients who remain symptomatic. In an experimental study, the introduction of an objective, saliva-based test significantly improved diagnostic accuracy for MNA and DDIs and led to more relevant treatment plans, including medication adjustments and counseling. Definitive MNA and DDI testing has the potential to impact how we evaluate follow-up patients with chronic disease and thereby improve patient outcomes and value in real-world settings.

## Supporting information

S1 File 04-AEGIS-2021 Protocol, cleaned. (PDF)

S2 File CONSORT_2025_editable_checklist. (DOCX)

S3 File S2_Data. (CSV)

S1 Appendix Virtual patient case development matrix. (DOCX)
