# Supplementary material for: Helping primary care providers recognize and respond to medication non-adherence and drug-drug interactions: A randomized-controlled clinical utility trial in a value-based care setting
Source: PLoS One. 2026 Mar 16;21(3):e0344906. doi: 10.1371/journal.pone.0344906 (PMC12991271; doi:10.1371/journal.pone.0344906)
Supplement: S1 File — (PDF) [file pone.0344906.s001.pdf]

CLINICAL STUDY PROTOCOL

Protocol Title: Measuring the Clinical Utility of Aegis's Chronic Disease Management Test among VillageMD Providers: A CPV® Randomized Controlled Trial

Protocol Number: 04-AEGIS-2021

Study Sponsor: QURE Healthcare
450 Pacific Ave, Suite 200 San Francisco, CA 94133 Phone +1 (415) 321-3388

Protocol Date: May 18, 2022

PROTOCOL ACCEPTANCE PAGE

Protocol Title: Measuring the Clinical Utility of Aegis's Chronic Disease Management Test among VillageMD Providers: A CPV® Randomized Controlled Trial

Protocol Number: 04

Protocol Date: May 18, 2022

Approvals

______________________________ May 18, 2022
Date

John W Peabody MD, PhD, FACP
Principal Investigator
President, QURE, LLC

______________________________ May 18, 2022
Czarlota Valdenor, MD Date
Project Lead
QURE, LLC

______________________________ May 18, 2022
Joel Galanter Date
Chief Legal Officer, Aegis Sciences Corp., Inc.

______________________________ May 18, 2022
John Torontow Date
Executive Vice President, VillageMD

REVISION HISTORY

Revision	Originator	Date Effective	Nature of Change
A			
B			
C			
D			

ACRONYMS

CDMT	Chronic Disease Management Testing
CPV®	Clinical Performance and Value® vignette
DDI	Drug-drug interaction
AF	Atrial Fibrillation
DM	Diabetes mellitus
HTN	Hypertension
COPD	Chronic Obstructive Pulmonary Disease
PCP	Primary care provider

STUDY SYNOPSIS

Protocol Title Measuring the Clinical Utility of Aegis's Chronic Disease Management Test among VillageMD Providers: A CPV® Randomized Controlled Trial

Background This study is the third amendment to the recently completed QURE 3 study (approved on October 19th, 2021) which measured the clinical utility of the Chronic Disease Management Test (CDMT) among US-based primary care physicians. The specific purpose of this study, hereon referred to as QURE Village, is to extend the findings of the original QURE 3 study and assess the clinical utility of the CDMT at selected VillageMD clinics nationwide.

VillageMD is a national leader in primary care that focuses on improving chronic disease management. They devote 90% of resources to mitigating the effects of chronic conditions such as heart failure or chronic obstructive pulmonary disease (COPD).

In the original QURE 3 study protocol, which is described in more detail below, we measured changes in prescribing practices using Clinical Performance and Value (CPV®) vignettes. The CPVs are a validated measurement tool that we used to demonstrate the enormous clinical variation in the management of chronic cardiometabolic diseases and with that we then assessed the impact of CDMT testing to improve physicians' clinical decision-making.

From the baseline data collected, we found significant variation in how physicians managed cardiometabolic diseases. In cases where patients were experiencing symptoms of uncontrolled disease due to medication non-adherence, physicians failed to identify non-adherence as the etiology. This failure in diagnosis led to an increase in unnecessary medication adjustments and referrals, prompting higher risk for adverse events. Furthermore, in patients with symptoms caused by drug-drug interactions (DDI), physicians only identified DDIs in 13% of patients. Finally, in patients who have disease progression despite adherence to medication and lack of DDIs, physicians continued with the patients' current medication despite worsening disease.

This amendment allows us to assess if a new set of primary care providers (PCP), who are particularly focused on chronic disease management, can change their practice after introduction to the CDMT. VillageMD is a rapidly growing group of highly coordinated and aligned PCPs who excel in patient care with multiple medical conditions. For VillageMD, findings from this study would represent a significant opportunity to improve their industry leading care services to better

address medication non-adherence, check for DDIs, and improving care for patients who have progressive disease.[1,2,3] QURE, under Aegis's sponsorship and with VillageMD's participation, will carry out a randomized control clinical trial among VillageMD primary care providers.

QURE Village extends the findings from QURE 3 by measuring the change in clinical practice using CPVs® at a specific, targeted primary care provider network. We will look for the same changes we found in QURE 3 to determine the impact of the CDMT on clinical decisions and patient outcomes at VillageMD.

Summary

Medication non-adherence in patients with chronic diseases is common, costly, and often underrecognized. With the new CDMT, an oral fluid-based non-invasive diagnostic test, the goal is to see if testing efficiently determines medication adherence and drug-drug interactions (DDIs) for patients with cardiometabolic multimorbidity. The test, produced by Aegis, detects and provides clinicians with adherence data on 150+ relevant medications across four major cardiometabolic conditions (coronary artery disease, heart failure, hypertension, diabetes, and COPD).

Factors contributing to poor medication adherence are myriad and are compounded by clinicians' lack of awareness to identify non-adherence and recognize DDIs (Marcum 2013). Conversely, DDIs also lead to non-adherence (Rugo 2021). All these signify a need for a simple, objective test that allows providers to recognize non-adherence and DDIs (Marcum 2013). Aegis is looking to make the CDMT available to more patients that can benefit from the test. Accordingly, Aegis has approached one of the nation's leading primary care models to determine whether VillageMD providers could change their practice in such a way that is beneficial to the most patients.

QURE Healthcare is a team of clinicians and researchers that conducts randomized-controlled trials using **Clinical Performance and Value (CPV®) patient simulations to generate high quality clinical utility evidence.** CPVs® are QURE's scientifically-validated measurement tool, first described in *JAMA*, 2000 and now used in scores of scientific investigations. In this research and derivative peer-reviewed publications, QURE's CPV® studies efficiently measure clinical practice patterns among active providers to determine if there is value of new technologies

---

[1] Peabody, J.; Schrecker, J; Heltsley, R.; Paculdo, D.; de Belen, E.; Tamondong-Lachica, D.; Acelajado, M.C.; Ouenes, O.; Kennedy, T.; Jeter, E. Randomized Trial to Improve Primary Care Patient Management and Patient Outcomes Using a Drug–Drug Interaction Test: Confirmation of the DECART Simulated Patient Clinical Utility Trial Results. Diagnostics 2021,11,1266. https://doi.org/10.3390/diagnostics11071266

[2] Peabody, J., Tran, M., Paculdo, D., Schrecker, J., Valdenor, C., & Jeter, E. (2018). Clinical Utility of Definitive Drug-Drug Interaction Testing in Primary Care. *Journal of clinical medicine*, *7*(11), 384. https://doi.org/10.3390/jcm7110384

[3] Peabody J, Acelajado MC, Robert T, Hild C, Schrecker J, Paculdo D, Tran M, Jeter E. Drug-Drug Interaction Assessment and Identification in the Primary Care Setting. J Clin Med Res. 2018 Nov;10(11):806-814. doi: 10.14740/jocmr3557w. Epub 2018 Oct 9. PMID: 30344815; PMCID: PMC6188027.

to payers.

QURE will generate unbiased clinical utility evidence experimentally for VillageMD, using the validated CPV® randomized-controlled trial (RCT) approach. More specifically, QURE will demonstrate how the CDMT results change clinical decisions made by practicing primary care providers at VillageMD and how these changes align with evidence-based care options for patients.

A key element in determining clinical utility is determining the use cases where the CDMT adds the most value. Because the clinical presentation and course for each CPV® patient can be precisely customized, QURE's validated patient simulation approach is uniquely positioned to explore multiple use cases and quantify the impact of the CDMT within specific use cases. These cases can be strategically crafted to provide VillageMD repeated opportunities to gather data on the use cases of most value for strategic, regulatory, and educational purposes. Based on the results of QURE 3, QURE, in collaboration with VillageMD clinical leadership, will update the cases to be used in QURE Village to present a new set of patients to the VillageMD providers.

Study Objectives

(1) To measure the variation in clinical decisions among practicing VillageMD primary care providers in the assessment, recognition, and adjustment of treatment due to medication non-adherence and DDIs in patients with chronic cardiometabolic diseases.
(2) To determine whether education about the Chronic Disease Management Test and provision of the Chronic Disease Management Test results (i.e., the intervention) improves the quality of care, as measured by evidence-based management decisions to recognize and manage medication non-adherence including DDIs and the chronic cardiometabolic conditions for which these medications are prescribed.
(3) To model whether receiving the Chronic Disease Management Test results leads to higher-value and/or lower-cost care decisions, including avoidance of invasive diagnostic tests or interventions, reduction of unneeded specialist referrals, increased diagnosis and treatment of drug-drug interactions or care decisions that are expected to reduce overall costs (e.g., appropriate and safe medication prescription that will reduce risk of cardiometabolic morbidity and mortality).
(4) To determine whether the Chronic Disease Management Test results differentially impact the quality of care for different patient types (use cases).
(5) To determine whether the targeted implementation of the Chronic Disease Management Test at VillageMD clinics could serve as a proof of concept for the remaining VillageMD primary care providers nationwide.

Study Design

The study is a prospective randomized cohort two-arm trial with six steps:

1) Enrollment: The study will enroll an estimated 166 practicing primary care providers, who practice at VillageMD clinics. An eligibility screener will be used to screen for eligibility (attached as Appendix A).
2) Provider survey: Once providers are enrolled in the study, they will be asked to complete an item questionnaire describing their practice, professional background and confidence in treating patients with cardiometabolic conditions.
3) Randomization: The 166 VillageMD providers will be randomized into two equally- sized arms: control group and intervention group.
4) CPVs® (First Round): Providers in both groups will complete three CPV® patient vignettes. Cases will be identical across the intervention and control arms. Cases are interactive and are presented on an online platform that is accessible via unique weblinks and any internet-connected computer.
5) Intervention education: For the intervention group, providers will receive educational materials describing the clinical validation and use cases of the Chronic Disease Management Test. The education materials are an orientation to the test, its use, the results directed to the participants that will adopt the CDMT (e.g., introduction slide deck, fact sheet, and/or case studies)
6) CPVs® (Second Round): All participating providers will then be asked to complete three additional CPV® patient simulations. The cases are again given to the doctors in a random order.

Study Instruments

There are two data collection instruments: 1) the provider questionnaire; and 2) the CPV® simulated patient cases.

The online provider questionnaire consists of 10-15 questions to better understand provider demographics, training, and practice environment. It will be administered once upon enrollment into the study.

The CPV® cases simulate typical patients with chronic cardiometabolic conditions and polypharmacy. In each case, providers will care for the simulated patient by indicating their preferred course of action to work-up, assess, and manage these patients. Providers will complete the cases in random order to cancel out any learning effects (which are known to be small); only the intervention group will receive CDMT results at the appropriate point in each round 2 CPV® case.

Study Interventions

Intervention-arm providers will receive two interventions:

- Educational materials
    - All intervention providers will first receive educational materials (e.g., a slide deck, webinar, fact sheet and/or case studies) on the Chronic Disease Management Test.
- CDMT results
    - All intervention providers will receive CDMT results at the appropriate point in each of the round 2 CPV® cases whether they select it or not.

Outcome Measures

- Baseline levels of variation in the work-up, recognition, and management of medication non-adherence and/or DDI in patients with more than one cardiometabolic condition and associated polypharmacy among all participants.
- Pre- and post-difference in the overall and the work-up, recognition, and management quality scores between control providers using standard of care diagnostic tools and intervention providers with access to CDMT results.
- Differences in evidence-based decisions, particularly prescribing decisions, made by intervention providers and control providers, while controlling for provider and practice characteristics, between rounds 1 and 2.
- Distinguishing between medication non-adherence, DDI, and full compliance with suboptimal medication treatment (inadequate dosing vs. ineffective medication).
- Difference in expected cost of care between control and intervention providers (Cost will be calculated by measuring differential rates of medical interventions/levels of care selected by each arm, and multiplying by Medicare reimbursement rates for these interventions, and/or by modeling the incidence of expected complications and calculating associated costs per above).
- Difference in the above cost and clinical quality metrics by three different use case types.

Population

Representative sample of practicing primary care providers in VillageMD clinics.

Provider Recruitment

Primary care provider participants will be contacted via an inclusive roster provided by VillageMD's leadership. Providers will include physicians, nurse practitioners and physician assistants. Potential participants will be contacted by email using a recruitment letter inviting them to participate. Participation is voluntary. All participants will be provided fair-market honoraria for their time.

We will maintain provider privacy and confidentiality in accordance with standard ethical practice standards. Accordingly, informed consent will be obtained by having participants read the consent form and provide their acceptance via an online voluntary consent process where users indicate their agreement to participate at the time they are enrolled.

Once informed consent is obtained, providers will be considered enrolled into the study. The study administrators will tell the participants that all data will be kept confidential and the appropriate information about the study will be disclosed to subjects prior to enrollment. There are adequate provisions to ensure subject privacy, and subjects are equitably selected to participate in the research.

Sample Size

The study will recruit 166 providers (83 per arm), with an expected sample size of 75 participants in each arm accounting for an estimated 10% attrition.

Study Hypothesis

1) Provider participants at baseline will not recognize that patients do not meet therapeutic goals due to medication non-adherence and show high levels of unwarranted variation in their work-up, recognition, and management of medication non-adherence in patients with more than one chronic cardiometabolic condition and associated polypharmacy in the CPV® patient simulations.

2) Providers will not recognize that patients do not meet therapeutic goals due to DDI and show high levels of unwarranted variation in their work-up, recognition, and management of medication non-adherence in patients with more than one chronic cardiometabolic condition and associated polypharmacy in the CPV® patient simulations.

3) Providers will not recognize that patients do not meet therapeutic goals due to ineffective therapy and show high levels of unwarranted variation in their work-up, recognition, and management of medication non-adherence in

patients with more than one chronic cardiometabolic condition and associated polypharmacy in the CPV® patient simulations.

4) The intervention group, those who receive educational materials about the CDMT and CDMT results in their round 2 patients, will improve their quality of care and have less practice variation when caring for their CPV® patients with more than one chronic cardiometabolic condition and associated polypharmacy, as measured by management decisions to recognize and/or manage medication non-adherence, DDI, and associated co-morbidities.

5) The additional information from the CDMT will result in better distinction between medication non-adherence and DDI and lead to better overall care including reduction in over-use of invasive diagnostic work-up and treatment and a reduction in cost in a subset of patients. The associated cost savings, particularly for those patients will come from obviating high-cost workup, avoidance of unneeded subspecialty referrals, increased diagnosis and treatment of drug-drug interactions and/or reducing the frequency or number of medical interventions ordered.

6) Of the nine simulated cases that will focus on patients with multiple chronic cardiometabolic conditions and polypharmacy, there will be a subset of use cases where the CDMT demonstrates greatest utility for patients.

7) Providers with access to the education materials and CDMT will diagnose medication non-adherence and potentially harmful DDIs more often than control providers.

Data Analysis

A pre-post analysis will be conducted to compare practice patterns and performance of the intervention versus the control group (statistically a difference in difference fixed effects analysis). This analysis uses the CPV® scores as the outcome variable and includes basic bivariate analysis to determine group differences followed by a difference-in-difference, fixed effects multi-variate regression analysis, controlling for provider characteristics, to identify significant differences across the two groups.

1. BACKGROUND INFORMATION AND RATIONALE

Medication non-adherence in patients with chronic cardiometabolic diseases is common, costly, and often underdiagnosed. Stroke, hypertension, heart disease, and diabetes account for over $450 billion in healthcare expenditures annually in the US (Lum 2020). Within these disease states, rates of polypharmacy often exceed 65% contributing to a significant increase in risk for adverse drug events and drug-drug interactions (DDI). With the new Chronic Disease Management Test (CDMT), a non-invasive diagnostic test, the goal is to see if testing efficiently determines medication adherence and drug-drug interactions (DDIs) for patients with cardiometabolic multimorbidity, defined by the WHO as the coexistence of two or more chronic diseases in the same individual. This oral fluid-based test, produced by Aegis, detects and provides clinicians with adherence and DDI data on 150+ relevant medications across four major cardiometabolic conditions (coronary artery disease, heart failure, hypertension, diabetes, and COPD).

There are multiple reasons for poor medication adherence from those related to patients (e.g., suboptimal health literacy), to those that are related to physicians (e.g., medications prescribed by other physicians or being unaware about the patient's adherence to prescribed medications), to those related to health care systems (e.g., limited access to follow-up care, absence of health information technology) (Brown 2013). Primary care providers absolutely must know whether their patients are complying with their prescribed medications to provide reliable and quality care. The factors that underlie non-adherence are underscored by how infrequently clinicians identify and diagnose nonadherence (Marcum 2013). DDIs are also unrecognized (Peabody 2018) and lead to non-adherence (Rugo 2021). All these factors signify a need for a simple, objective intervention that allows providers to recognize non-adherence and DDI (Marcum 2013). CDMT offers the advantage of a medication adherence assessment method that is accurate, valid, and sensitive to change. Furthermore, the CDMT has the ability to detect drug interactions when DDI testing is included, which leads to more non-adherence, drug toxicity, or loss of efficacy of the co-administered medication, and other medication-related problems. Accordingly, Aegis has approached one of the nation's leading primary care models to determine whether VillageMD providers could change their practice in such a way that is beneficial to the most patients.

The measurement of patient medication adherence and use of interventions to improve adherence are rare in routine clinical practice. For this reason, medication adherence has been called the "next frontier in quality improvement" and is an important part of cardiovascular outcomes research (Ho 2009). With this study, we are providing objective and meaningful data that will provide a measure of medication adherence and assess the impact of non-adherence recognition in provider practice.

This study is the third amendment to the ongoing QURE 3 study (approved on October 19th, 2021). The specific purpose of this study, hereon referred to as QURE Village, is to extend the findings of the original QURE 3 study and assess the clinical utility of the Chronic Disease Management Test (CDMT) at selected VillageMD clinics nationwide.

VillageMD is a national leader in primary care that focuses on improving chronic disease management. They devote 90% of resources to mitigating the effects of chronic conditions such as heart failure or chronic obstructive pulmonary disease (COPD).

In the original QURE 3 study protocol, which is described in more detail below, measured changes in prescribing practices using Clinical Performance and Value (CPV®) vignettes. the CPVs are a validated measurement tool that we used to demonstrate the enormous clinical variation in the management of chronic cardiometabolic diseases and with that we then assessed the impact of CDMT testing to improve physicians' clinical decision-making.

We found from the baseline data collected, significant variation in how physicians managed cardiometabolic diseases. In cases where patients were experiencing symptoms of uncontrolled disease due to medication non-adherence, physicians failed to identify non-adherence as the etiology which led to an increase in unnecessary medication adjustments and referrals, prompting higher risk for adverse events. Furthermore, in patients with symptoms caused by drug-drug interactions (DDI), physicians only identified DDIs in 13% of patients. Finally, in patients who have disease progression despite adherence to medication and lack of DDIs, physicians continued with the patients' current medication despite worsening disease.

This amendment allows us to ask a new set of primary care providers (PCP) that are particularly focused on chronic disease management, if they can change their practice after introducing them to the CDMT. VillageMD is a rapidly growing group of highly coordinated and aligned PCPs who excel in care of patients with multiple medical conditions. For VillageMD, findings from this study would represent a significant opportunity to improve their industry leading care services to better address medication non-adherence, check for DDIs, and improving care for patients who have progressive disease.[4,5,6] QURE, under Aegis's sponsorship and with VillageMD's participation, will carry out a randomized control clinical trial among their VillageMD primary care providers.

[4] Peabody, J.; Schrecker, J; Heltsley, R.; Paculdo, D.; de Belen, E.; Tamondong-Lachica, D.; Acelajado,M.C.; Ouenes, O.; Kennedy, T.; Jeter,E. Randomized Trial to Improve Primary Care Patient Management and Patient Outcomes Using a Drug–Drug Interaction Test: Confirmation of the DECART Simulated Patient Clinical Utility Trial Results. Diagnostics 2021,11,1266. https://doi.org/10.3390/diagnostics11071266

[5] Peabody, J., Tran, M., Paculdo, D., Schrecker, J., Valdenor, C., & Jeter, E. (2018). Clinical Utility of Definitive Drug-Drug Interaction Testing in Primary Care. *Journal of clinical medicine*, *7*(11), 384. https://doi.org/10.3390/jcm7110384

[6] Peabody J, Acelajado MC, Robert T, Hild C, Schrecker J, Paculdo D, Tran M, Jeter E. Drug-Drug Interaction Assessment and Identification in the Primary Care Setting. J Clin Med Res. 2018 Nov;10(11):806-814. doi: 10.14740/jocmr3557w. Epub 2018 Oct 9. PMID: 30344815; PMCID: PMC6188027.

QURE Village extends the findings from QURE 3 by measuring the change in clinical practice using CPVs® at a specific, targeted primary care provider network. We will look for the same changes we found in QURE 3 to determine the impact of the CDMT on clinical decisions and patient outcomes at VillageMD.

QURE Healthcare is a team of clinicians and researchers that conducts randomized-controlled trials using **Clinical Performance and Value (CPV®) patient simulations to generate high quality clinical utility evidence.** CPVs® are QURE's scientifically-validated measurement tool, first described in *JAMA*, 2000 and now used in scores of scientific investigations. In this research and derivative peer-reviewed publications, QURE's CPV® studies efficiently measure clinical practice patterns among active providers to determine if there is value of new technologies to payers.

QURE's CPVs® are a unique and scalable tool that standardizes practice measurement by having all providers care for the same (virtual) patient (*Annals of Int Med, 2004*). With all providers taking care of the same patients, QURE generates unbiased data that yields powerful insights into clinical decision making and how these decisions change with the introduction of a new product or solution (*Health Policy and Planning*, 2009). Data from the CPVs® can quickly demonstrate the clinical utility of a solution, be published in the peer-reviewed literature, and positively impact coverage and reimbursement decisions (*PLoS One, 2013* and 2016). QURE CPVs® do this at a fraction of the time and cost of other data studies.

QURE will generate unbiased clinical utility evidence experimentally for VillageMD, using the validated CPV® randomized-controlled trial (RCT) approach. More specifically, QURE will demonstrate in the study how the Chronic Disease Management Test results change clinical decisions made by practicing primary care providers and how these changes align with evidence-based care options for patients.

A key element in determining clinical utility is determining the use cases where the Chronic Disease Management Test adds the most value. Because the clinical presentation and course for each CPV® patient can be precisely customized, QURE's validated patient simulation approach is uniquely positioned to explore multiple use cases and quantify the impact of the Chronic Disease Management Test within specific use cases. These cases can be strategically crafted to provide Aegis repeated opportunity to gather data on the use cases of most value for strategic, regulatory, and educational purposes. Based on the results of QURE 3, QURE will update the cases to present a new set of patients to the VillageMD providers.

2. STUDY DESIGN

The study is an extension of QURE 3's patient simulation-based randomized-controlled study design which recruited a representative national sample of physicians. This QURE Village study will select a targeted and inclusive group of primary care providers in VillageMD clinics and assign them to either the control or the intervention group. All providers will then care for simulated patients via CPVs®.

Providers in both groups will complete, in random order, three CPV® patient simulations to determine their current practice when assessing patients facing medication non-adherence and DDIs. Cases will be identical across the intervention and control arms. Cases are interactive and are presented on an online platform that is accessible via unique weblinks and any internet-connected computer.

After the initial set of CPVs® are completed, intervention group providers will receive educational materials describing the clinical validation and use cases of the CDMT. The education materials are an orientation to the test, its use, and sample results directed to the participants who will adopt the CDMT (e.g., introduction slide deck, fact sheet, and/or case studies).

After receiving the CDMT education materials, intervention group providers will be asked to complete another set of CPVs®, this time, they'll be able to access the CDMT and its results and use it to diagnose and treat their patient. Control group providers will neither access the CDMT educational material nor be able to use its results in their routine care for their simulated patient.

2.1 Study Instruments

There are two data collection instruments: 1) the provider questionnaire; and 2) the CPV® simulated patient cases.

The online provider questionnaire consists of 10-15 questions to better understand provider demographics, training and practice environment. It will be administered once, upon enrollment into the study.

The CPV® cases will be designed to simulate typical patients with chronic cardiometabolic conditions and polypharmacy. In each case, providers will care for the simulated patient by indicating their preferred course of action to work- up, assess, and manage these patients. Providers will complete the cases in random order to cancel out any learning effects (which are known to be small), and only the intervention group will receive CDMT results at the appropriate point in each round 2 CPV® case whether they select it or not. All told, nine cases

will be developed, and any given provider will randomly be assigned to two rounds of three cases. Intervention will occur between the two rounds.

CPV® vignettes will be designed based on a 3 x 3 matrix of chronic cardiometabolic diseases and common drivers for non-adherence and potential drug-drug interactions.

	**Variant A:** Not at goal Test confirms medication non-adherence, no DDI	**Variant B:** Not at goal Therapeutic failure due to DDI and/or Patient at Risk for ADE	**Variant C:** Not at goal, DDI suspected Test indicates adherence to medications as prescribed, no DDI
**COPD**	Case 1A	Case 1B	Case 1C
**HF/AF**	Case 2A	Case 2B	Case 2C
**DM/HTN**	Case 3A	Case 3B	Case 3C

2.2 Study Interventions

Intervention-arm providers will receive two interventions:

- Educational materials
    - All intervention group providers will first receive educational materials (e.g., a slide deck, webinar, fact sheet and/or case studies) on the CDMT.
- CDMT results
    - All intervention group providers will receive CDMT results at the appropriate point in each of the round 2 CPV® cases whether they select it or not.

3. STUDY OBJECTIVES

This study will test the improvement in quality of provider care decisions concerning medication non-adherence among patients with chronic cardiometabolic conditions using a well-validated patient-simulation based measurement approach, CPVs®, to assess the following:

(1) To measure the variation in clinical decisions among practicing VillageMD primary care providers in the assessment, recognition, and adjustment of treatment due to medication non-adherence and DDI in patients with chronic cardiometabolic diseases.

(2) To determine whether education about the Chronic Disease Management Test and provision of Chronic Disease Management Test results (i.e., the intervention) improves the quality of care, as measured by evidence-based management decisions to recognize and manage medication non-adherence including DDIs and the chronic cardiometabolic

conditions for which these medications are prescribed.

(3) To model whether receiving Chronic Disease Management Test results leads to higher-value and/or lower-cost care decisions, including avoidance of invasive diagnostic tests or interventions, reduction of unneeded specialist referrals, increased diagnosis and treatment of drug-drug interactions, or care decisions that are expected to reduce overall costs (e.g., appropriate and safe medication prescription that will reduce risk of cardiovascular morbidity and mortality).

(4) To determine whether the Chronic Disease Management Test results differentially impact the quality of care for different patient types (use cases).

(5) To determine whether the targeted implementation of the Chronic Disease Management Test at VillageMD clinics could serve as a proof of concept for the remaining VillageMD primary care providers nationwide.

3.1 Outcome Measures

- Baseline levels of variation in the work-up, recognition, and management of medication non-adherence and/or DDI in patients with more than one chronic cardiometabolic conditions and associated polypharmacy among all participants.
- Pre- and post-difference in the overall and the work-up, recognition and management quality scores between control providers using standard of care diagnostic tools and intervention providers with access to CDMT results.
- Differences in evidence-based decisions, particularly prescribing decisions, made by intervention providers and control providers, while controlling for provider and practice characteristics, between rounds 1 and 2.
- Distinguishing between medication non-adherence, DDI, and full compliance with suboptimal medication treatment (inadequate dosing vs. ineffective medication).
- Difference in expected cost of care between control and intervention providers (Cost will be calculated by measuring differential rates of medical interventions/levels of care selected by each arm, and multiplying by Medicare reimbursement rates for these interventions, and/or by modeling the incidence of expected complications and calculating associated costs per above).
- Difference in the above cost and clinical quality metrics and cost measures by three different use case types.

4. ELIGIBILITY CRITERIA

a. Description of subjects

Practicing providers will be the study subjects with the following eligibility criteria.

Inclusion Criteria

Subjects must meet the following criteria to be enrolled in the study:

1. Licensed primary care provider (PCP) (MD, DO, NP, PA) currently

practicing in the following areas:
   a) Internal medicine
   b) Family medicine
2. Have practiced as a PCP in internal or family medicine for greater than 2 but less than 30 years
3. Community / non-academic based practice setting
4. ≥ 40 patients under care weekly
5. Commonly treats patients with atrial fibrillation, coronary artery disease, congestive heart failure, diabetes, hypertension, COPD, and hyperlipidemia
6. Practicing in the U.S.
7. English-speaking
8. Access to the internet
9. Informed and voluntarily consented to be in the study

b. Subject Screening and Enrollment

Primary care provider participants will be contacted via an inclusive roster provided by VillageMD's leadership. Providers will include physicians, nurse practitioners and physician assistants. Potential participants will be contacted by email using a recruitment letter inviting them to participate. Participation is voluntary. All participants will be provided fair-market honoraria for their time.

We will maintain provider privacy and confidentiality in accordance with standard ethical practice. Informed consent will be obtained by having providers read the consent form and provide their acceptance via an online voluntary consent process where users indicate their agreement to participate at the time they are enrolled. We believe the voluntary consent process is sufficient for this study given the scientific sophistication of the study population and the very low risks of participation.

Providers will be considered enrolled into the study once informed consent is obtained.

c. Subject Withdrawal

If a participant fails to complete 3 CPV® vignettes per round, they will be withdrawn from the study and replaced with another provider. Any CPVs® from withdrawn subjects will be excluded from the analysis.

i. Withdrawal Criteria

Reasons for study withdrawal may include but are not limited to:

- Non-compliance with study procedures.
- Provider's right to withdraw consent at any time during the study with or without stated reason.

ii. Documentation of Withdrawal of Subjects
The reason for withdrawal of any provider from the study will be appropriately documented.

5. TREATMENT OF SUBJECTS

The subjects are primary care providers, presenting minimal risk to participation that is likely no different from the probability and magnitude of physical or psychological harm that is normally encountered in the daily lives. Possible risk would be loss of confidentiality and revelation of their case results, which will only be available to study administrators. Participants (providers) will be informed of these risks and asked to provide consent before participation. Participation is strictly voluntary. Subjects will be appropriately compensated for their time participating in the study.

6. STUDY PROCEDURES

At enrollment, each eligible provider who agrees to participate will be assigned a unique subject identification number for internal tracking of completion and data collection. Only the study team will have access to the subject's identity. The subject's name or other identifying information will not be used in analysis or reporting.
The main study procedures or assessments will include but are not limited to: collection of demographic and practice information during the screening process and completion of CPV® cases.

6.1 Pre-Screening and Eligibility

The evaluations for inclusion and exclusion criteria will include professional licensure and clinical volume, as described in Section 4 above.

6.2 Completion of Questionnaire

Enrolled providers will then be asked to complete a questionnaire describing their practice and professional background. The questionnaire will consist of 10-15 questions to better understand provider demographics, training and practice environment. It will be administered immediately upon enrollment into the study.

6.3 Completion of CPV® cases

CPV® cases will be designed to simulate typical patients with chronic cardiometabolic conditions and polypharmacy presenting in clinical situations. In each case, providers will answer questions about their preferred course of action to work-up, assess, and manage patients. Intervention group providers only will also receive educational materials upon the CDMT prior to their CPV® cases, and during their post-intervention CPV® cases, will receive CDMT results at the appropriate point in each round 2 CPV® case whether they select it or not.

7. STUDY DATA COLLECTION

Data entry and resolution will be performed in real-time concurrent with CPV® data collection. All data will be linked to providers via a unique identification number; only the study team will have access to their identities. Electronic data files will be password protected. Periodic audits will ensure data protection procedures are being followed. No providers will be mentioned by name in any data output.

8. STATISTICAL ANALYSIS

a. Study Hypotheses

1. Provider participants at baseline will not recognize that patients do not meet therapeutic goals due to medication non-adherence and show high levels of unwarranted variation in their work-up, recognition, and management of medication non-adherence in patients with more than one chronic cardiometabolic conditions and associated polypharmacy using in the CPV® patient simulations.
2. Providers will not recognize that patients do not meet therapeutic goals due to DDI and show high levels of unwarranted variation in their work-up, recognition, and management of medication non-adherence in patients with more than one chronic cardiometabolic conditions and associated polypharmacy using in the CPV® patient simulations.
3. The intervention group, those who receive educational materials about the CDMT and CDMT results in their round 2 patient, will improve their quality of care and have less practice variation when caring for their CPV® patients with more than one chronic cardiometabolic conditions and associated polypharmacy, as measured by management decisions to recognize and/or manage medication non-adherence, DDI, and associated co-morbidities.
4. The additional information from the CDMT will result in better distinction between medication non-adherence and DDI and lead to better overall care including reduction in over-use of invasive diagnostic work-up and treatment, and a reduction in cost in a subset of patients. The associated cost savings, particularly for those patients will come from obviating high-cost workup, avoidance of unneeded subspecialty referrals, increased diagnosis and treatment of drug-drug interactions and/or reducing the frequency or number of medical interventions ordered.
5. Of the nine simulated cases that will focus on patients with multiple chronic cardiometabolic conditions and polypharmacy, there will be a subset of use cases where the CDMT demonstrates greater utility.
6. Providers with access to the education materials and CDMT will diagnose

medication non-adherence and potentially harmful DDIs more often than control providers.

b. Planned Evaluations

Baseline Data Analysis

At baseline parametric and non-parametric estimates of differences will be calculated between participants pre- and post-intervention to examine differences in assessment score. Differences will be adjusted using multivariate and logistic regression models constructed to include age, gender, specialty training, facility capabilities, clinical volume, practice size, and other practice characteristics, and by patient characteristics such as type of payers. Non-parametric estimates of differences in care utilization will be calculated and adjusted for provider, practice and patient characteristics described above.

Summary tables for each group will be generated for the following:

- Practice setting (private, community, etc.)
- Practice size
- Average weekly hours in practice
- Percent of patients covered by
    - Medicare
    - Commercial/Private
    - Medicaid
    - Self-pay / Uninsured
    - Other
- Mean (+/- 2 SD)
    - Years in practice
    - Weekly hours in practice
    - Age
    - Number of hours of training in the last 12 months

Longitudinal Data Analysis

Primary Outcome Variables:

- Baseline variation in care among providers using standard of care tools, as measured by CPV® patient simulations
- In the intervention group, change in the overall and the diagnostic and treatment evidence-based quality scores and reductions in variation when caring for the CPV® simulated patient with the introduction of CDMT testing.

- Changes in evidence-based management medication decisions (treatment) based on patient's CDMT result.
- Distinguishing between medication non-adherence, DDI, and full compliance with suboptimal medication treatment (inadequate dosing vs. ineffective medication).
- Change in cost of chronic disease-related care. (This cost is modeled in part by measuring differential rates of work up/interventions/levels of care selected by each arm and multiplying by average Medicare reimbursement rates for these workups/interventions/ levels of care, and/or by modeling the incidence of expected complications and calculating associated costs per above).
- Difference in the above cost and clinical quality metrics and cost measures by three different use case types.

Secondary Outcome Variables:

- Calculation of the change in the expected cost and clinical quality metrics for each type simulated patient case (use case).

Main Explanatory Variable:

- Intervention status (exposure to intervention)

We will control for all potential confounders, including:

- Age
- Gender
- Years in practice
- Practice location
- Type of location (urban/suburban vs. rural)
- Weekly hours in clinical practice
- Number of providers in practice
- Avg. type of coverage of patients (Medicare, Medicaid, commercial, etc.)
- Participation in care quality incentive programs
- Percentage of patients that have chronic cardiometabolic disease

We will model CPV® scores as a continuous outcome variable in a multiple linear regression model, which controls for all potential confounders listed above.

Our regression model is as follows, where CPV® is the score for each provider and INTERV indicates intervention group and CASE indicates the order in which the provider took the case (first, second, third, fourth).

Y_{it} = CPV® score of provider *i*, at time *t*

$Y_{it} \sim Normal(\mu_{ijt}, \sigma^2)$

$\mu_{ijt} = \beta_0 + \beta_1 CASE + \beta_2 INTERV + \beta_3 CASE \times INTERV$ + provider-practice variables

For binary outcome variables, we will use a logistic regression model. Our regression is as follows, where INTERV indicates intervention group and TIME indicates time period (by week). For outcomes with a Poisson distribution, the distributional assumption would be changed from

Bernoulli to Poisson and the link would be changed from logit to log. Otherwise, the basic modeling and analysis strategy will remain the same.

Y_{it} = appropriate therapy *t*, provider *i*

p_{it} = probability of an outcome for time *t*, provider *i*

$\text{logit}(E[Y_{it}]) = \beta_0 + \beta_1 CASE + \beta_2 INTERV + \beta_3 TIME \times INTERV$
+ provider-practice variables

For count outcome variables we will use log linear regression (i.e., using a log link in the analysis). Cluster re-sampled bootstrapping will be used to check model distributional assumptions.

9. ADMINISTRATIVE CONSIDERATIONS

a. Study Compliance

The study will be conducted in compliance with this protocol, principles of ICH-E6, GCP and the Declaration of Helsinki and all applicable national regulations governing clinical trials.

b. Protected Subject Information

A copy of the IRB approved informed consent may be audited. The investigator or designee **must** explain to each subject the purpose and nature of the study, the study procedures, the possible adverse effects and all other elements of consent as defined in 21CFR §50. In accordance to individual local and national subject privacy regulations, the investigator or designee **must** explain to each subject prior to screening that for the evaluation of study results, the subject's protected information obtained during the study may be shared with QURE Healthcare and its designees, regulatory agencies and IECs/IRBs. QURE Healthcare and Aegis will not use the subject's protected information or disclose it to a third party without applicable subject authorization. It is the investigator's or designee's responsibility to obtain a written permission to use protected information from each subject. Any data collected from a subject prior to withdrawal will not be used in the analysis of study results.

c. Retention of Records
The files of study subjects shall be retained in accordance with national legislation and the maximum period of time permitted by QURE Healthcare. QURE Healthcare will maintain records and documents on-site.

d. Confidentiality and Publication Policy
For publications, authorship will be determined according to the generally accepted principles of authorships and by mutual agreement.

REFERENCES

Lum MV, Cheung MYS, Harris DR, Sakakibara BM. A scoping review of polypharmacy interventions in patients with stroke, heart disease and diabetes. Int J Clin Pharm. 2020;42(2):378-392. doi:10.1007/s11096-020-01028-x

Marcum ZA, Sevick MA, Handler SM. Medication nonadherence: a diagnosable and treatable medical condition. JAMA. 2013;309(20):2105-2106. doi:10.1001/jama.2013.4638

Rugo, HS, et al. Abstract PS10-09: Real-world analysis of concomitant medication use with potential drug-drug interactions (DDI) in patients with metastatic breast cancer (MBC) treated with cyclin dependent kinase (CDK) 4/6 inhibitors. Cancer Res February 15, 2021 (81) (4 Supplement) PS10-09; DOI: 10.1158/1538-7445.SABCS20-PS10-09

Ho PM, Bryson CL, Rumsfeld JS. Medication adherence: its importance in cardiovascular outcomes. Circulation. 2009 Jun 16;119(23):3028-35. doi: 10.1161/CIRCULATIONAHA.108.768986. PMID: 1952834

Brown MT, Bussell JK. Medication adherence: WHO cares? Mayo Clin Proc. 2011;86(4):304-314. doi:10.4065/mcp.2010.0575

Peabody JW, Luck J, Glassman P, Dresselhaus TR, Lee M. Comparison of vignettes, standardized patients, and chart abstraction: a prospective validation study of 3 methods for measuring quality. JAMA. 2000 Apr 5;283(13):1715-22. doi: 10.1001/jama.283.13.1715. PMID: 10755498.

Peabody J, Luck J, Glassman P, et al. Measuring the quality of physician practice by using clinical vignettes: a prospective validation study. Ann Intern Med. 2004 Nov; 141(10):771-780.

Solon, Orville, et al. "A novel method for measuring health care system performance: experience from QIDS in the Philippines." Health policy and planning 24.3 (2009): 167-174.

Burgon TB, Cox-Chapman J, Czarnecki C, Kropp R, Guerriere R, Paculdo D, Peabody, JW. "Engaging Primary Care Providers to Reduce Unwanted Clinical Variation and Support ACO Cost and Quality Goals: A Unique Provider-Payer Collaboration." Population Health Management. 2018/Oct 17 online ahead of print: http://doi.org/10.1089/pop.2018.0111

Peabody, John W., Vibeke Strand, Riti Shimkhada, Rachel Lee, and David Chernoff. "Impact of Rheumatoid Arthritis Disease Activity Test on Clinical Practice." PLoS ONE. 2013; 8.5: 1-7.

Peabody, John, et al. "Clinical utility of a comprehensive, whole genome CMA testing platform in pediatrics: a prospective randomized controlled trial of simulated patients in physician practices." PloS one 11.12 (2016): e0169

Peabody J, Acelajado MC, Robert T, Hild C, Schrecker J, Paculdo D, Tran M, Jeter E. Drug-Drug Interaction Assessment and Identification in the Primary Care Setting. J Clin Med Res. 2018 Nov;10(11):806-814. Doi: 10.14740/jocmr3557w. Epub 2018 Oct 9. PMID: 30344815; PMCID: PMC6188027
