# Supplementary material for: Helping primary care providers recognize and respond to medication non-adherence and drug-drug interactions: A randomized-controlled clinical utility trial in a value-based care setting
Source: PLoS One. 2026 Mar 16;21(3):e0344906. doi: 10.1371/journal.pone.0344906 (PMC12991271; doi:10.1371/journal.pone.0344906)
Supplement: S1 Appendix — (DOCX) [file pone.0344906.s004.docx]

**S1 Appendix.** Virtual Patient Case Development Matrix

|  |  | **CASE VARIANTS** | | |
| --- | --- | --- | --- | --- |
|  |  | **Variant A**  Test confirms medication non-adherence (MNA)  No drug-drug interaction (DDI) | **Variant B**  Not meeting treatment goal  Patient at risk for ADE or therapeutic failure due to DDI | **Variant C**  Not meeting treatment goal; MNA, DDI suspected  Test indicates adherence to medication as prescribed and no DDI |
| **Case Types** | **Case 1**  Heart failure with reduced ejection fraction (HFrEF) | **Case 1A**  See simulated patient details below | **Case 1B**  See simulated patient details below | **Case 1C**  See simulated patient details below |
|  | **Case 2**  HFpEF  (Heart failure with preserved ejection fraction) | **Case 2A**  See simulated patient details below | **Case 2B**  See simulated patient details below | **Case 2C**  See simulated patient details below |
|  | **Case 3**  COPD | **Case 3A**  See simulated patient details below | **Case 3B**  See simulated patient details below | **Case 3C**  See simulated patient details below |

## **Case 1A**

- **Demographics**: 67/F with progressive failure symptoms (orthopnea, easy fatigability) on maximal GDMT dose of bisoprolol
- **Comorbid** **illnesses**: HFrEF, DM, chronic venous insufficiency, dyslipidemia, overweight, depression, CKD
- **Medications**: bisoprolol, sacubitril/valsartan, metformin/empagliflozin, atorvastatin, escitalopram, furosemide PRN
- **Risk** **factors**: age, polypharmacy, multiple co-morbidity, elevated LDL, overweight, smoker

## **Case 1B**

- **Demographics**: 69/M with known HF (on metoprolol succinate, valsartan) who presents with persistent failure symptoms (edema, shortness of breath, and easy fatigability). On history he has a recent urgent care visit for knee pain (has osteoarthritis) – was prescribed celecoxib. He self-medicated with ibuprofen for knee pain also (has used his son’s medications in previous occasions)
- **Comorbid** **illnesses**: heart failure with reduced ejection fraction (HFrEF), hypertension, osteoarthritis, depression, BPH
- **Medications**: metoprolol succinate, valsartan, rosuvastatin, furosemide PRN for edema, tamsulosin
- **Risk factors**: age, polypharmacy, overweight, ethnic minority, multiple co-morbidity

## **Case 1C**

- **Demographics**: 64/F on follow-up after ED visit. Had progressive shortness of breath, exertional dyspnea, and cough with occasional sputum production (whitish, no change in character) over the past month; no fever, no bipedal edema. Seen at ED 1 week ago, managed as HF decompensation: beta blockers put on hold and prescribed short-course diuretics. Currently still with cough and shortness of breath. PE: tachycardic, wheezing on auscultation but no rales; no neck vein engorgement, no bipedal edema.
- **Comorbid illnesses**: HFrEF from IHD diagnosed 4 years ago, COPD Group B diagnosed 4 years ago; hypertension, newly diagnosed depression, bilateral knee osteoarthritis
- **Medications**:
  - Cardiac meds: bisoprolol (on hold), sacubitril/valsartan (optimal dose) atorvastatin, aspirin
  - For COPD: Fluticasone proprionate/salmeterol 250/50 mcg 1 puff BID, albuterol PRN
  - Others: Recently started taking kava supplements to help with sleep; Takes NSAIDs sporadically for knee pain
- **Risk factors**: ethnic minority, polypharmacy

## **Case 2A**

- **Demographics**: 78/F on follow-up, with poorly controlled hypertension on telmisartan, lives at home alone. On evaluation, patient has mild depression (likely cause of non-adherence)
- **Comorbid illnesses**: HTN, CKD, diabetes, HFpEF, GERD, depression
- **Medications**: telmisartan, carvedilol, pravastatin, sitagliptin/metformin, omeprazole
- **Risk factors**: older age, polypharmacy, multi-morbidity, poor social support, elevated LDL, ethnic minority

## **Case 2B**

- **Demographics**: 61/M, African American ancestry, presenting with fatigue
- **Comorbid illnesses**: HFpEF, hypertension, diabetes, low back pain, dyslipidemia, smokes 1 pack of cigarettes/day, carbuncle
- **Medications**: losartan, metoprolol, metformin, glipizide, ibuprofen PRN, simvastatin, trimethoprim/sulfamethoxazole
- **Risk factors**: age, ethnic minority, multiple co-morbidity, recent ED visit for skin and soft tissue infection, reduced coordination of care due to use of multiple providers

## **Case 2C**

- **Demographics**: 67/F complaining of headaches, with poorly controlled hypertension
- **Comorbid illnesses**: HTN, DM, obesity, HFpEF
- **Medications**: lisinopril (optimal dose), bisoprolol (optimal dose), metformin, linagliptin, rosuvastatin, supplement for wellness containing elderberry
- **Risk factors**: obesity, lower socioeconomic status, intake of herbal supplements, polypharmacy

## **Case 3A**

- **Demographics**: 68/M on follow-up for COPD Group B. Reports recent increase in cough and sputum production. Elevated BP 158/90.
- **Comorbid illnesses**: hypertension, low back pain, GERD, depression (contributes to non-adherence), dyslipidemia
- **Medications**: tiotropium 2.5ug 2 puffs once daily, albuterol 90 mcg PRN, losartan 50mg OD, simvastatin, omeprazole, diclofenac gel
- **Risk factors**: polypharmacy, multiple co-morbidity, lower socioeconomic status, higher co-pay

## **Case 3B**

- **Demographics**: 71/F with 5-day history of shortness of breath. Describes increased coughing and shortness of breath 5 days ago, initially relieved by albuterol inhaler, but reports persistent feeling of tiredness, like she just ran 4 blocks nonstop. PE and workup showed sinus tachycardia, BP elevated from baseline at 146/90, others unremarkable.
- **Comorbid illnesses**: COPD Group B, DM, dyslipidemia), hypertension, low back pain (on cyclobenzaprine)
- **Medications**: albuterol 90mcg, tiotropium 2.5 ug 2 puffs OD, canagliflozin/metformin, linagliptin, lisinopril, carvedilol, atorvastatin, cyclobenzaprine, multivitamin
- **Risk factors**: age, polypharmacy, multiple co-morbidity, ethnic minority

## **Case 3C**

- **Demographics**: 66/M on follow-up for COPD Group B. Reports persistent symptoms on current therapy. Had just shifted from umeclidinium to tiotropium 1 month ago due to difficulty using umeclidinium. Also started on sumatriptan 5 days ago because of moderate-severe acute migraine attacks unrelieved by NSAIDs/acetaminophen.
- **Comorbid illnesses**: Hypertension (controlled), BPH, dyslipidemia, migraine headache
- **Medications**: albuterol 90mcg PRN, tiotropium inhalation spray 2.5mcg 1 puff OD, valsartan, amlodipine, tamsulosin, sumatriptan, atorvastatin, multivitamins
- **Risk factors**: polypharmacy, multiple co-morbidity
